# Research progress on the microbial metabolism and transport of polyamines and their roles in animal gut homeostasis

**DOI:** 10.1186/s40104-025-01193-x

**Published:** 2025-04-15

**Authors:** Chong Zhang, Yongkang Zhen, Yunan Weng, Jiaqi Lin, Xinru Xu, Jianjun Ma, Yuhong Zhong, Mengzhi Wang

**Affiliations:** 1 Laboratory of Metabolic Manipulation of Herbivorous Animal Nutrition, College of Animal Science and Technology, Yangzhou University, Yangzhou, 225009 China; 2 State Key Laboratory of Sheep Genetic Improvement and Healthy Production, Xinjiang Academy of Agricultural Reclamation Sciences, Shihezi, 832000 China

**Keywords:** Gut homeostasis, Gut microbiota, Polyamines, Spermidine

## Abstract

Polyamines (putrescine, spermidine, and spermine) are aliphatic compounds ubiquitous in prokaryotes and eukaryotes. Positively charged polyamines bind to negatively charged macromolecules, such as nucleic acids and acidic phospholipids, and are involved in physiological activities including cell proliferation, differentiation, apoptosis and gene regulation. Intracellular polyamine levels are regulated by biosynthesis, catabolism and transport. Polyamines in the body originate from two primary sources: dietary intake and intestinal microbial metabolism. These polyamines are then transported into the bloodstream, through which they are distributed to various tissues and organs to exert their biological functions. Polyamines synthesized by intestinal microorganisms serve dual critical roles. First, they are essential for maintaining polyamine concentrations within the digestive tract. Second, through transcriptional and post-transcriptional mechanisms, these microbial-derived polyamines modulate the expression of genes governing key processes in intestinal epithelial cells—including proliferation, migration, apoptosis, and cell–cell interactions. Collectively, these regulatory effects help maintain intestinal epithelial homeostasis and ensure the integrity of the gut barrier. In addition, polyamines interact with the gut microbiota to maintain intestinal homeostasis by promoting microbial growth, biofilm formation, swarming, and endocytosis vesicle production, etc. Supplementation with polyamines has been demonstrated to be important in regulating host intestinal microbial composition, enhancing nutrient absorption, and improving metabolism and immunity. In this review, we will focus on recent advances in the study of polyamine metabolism and transport in intestinal microbes and intestinal epithelial cells. We then summarize the scientific understanding of their roles in intestinal homeostasis, exploring the advances in cellular and molecular mechanisms of polyamines and their potential clinical applications, and providing a rationale for polyamine metabolism as an important target for the treatment of intestinal-based diseases.

**Graphical Abstract:**

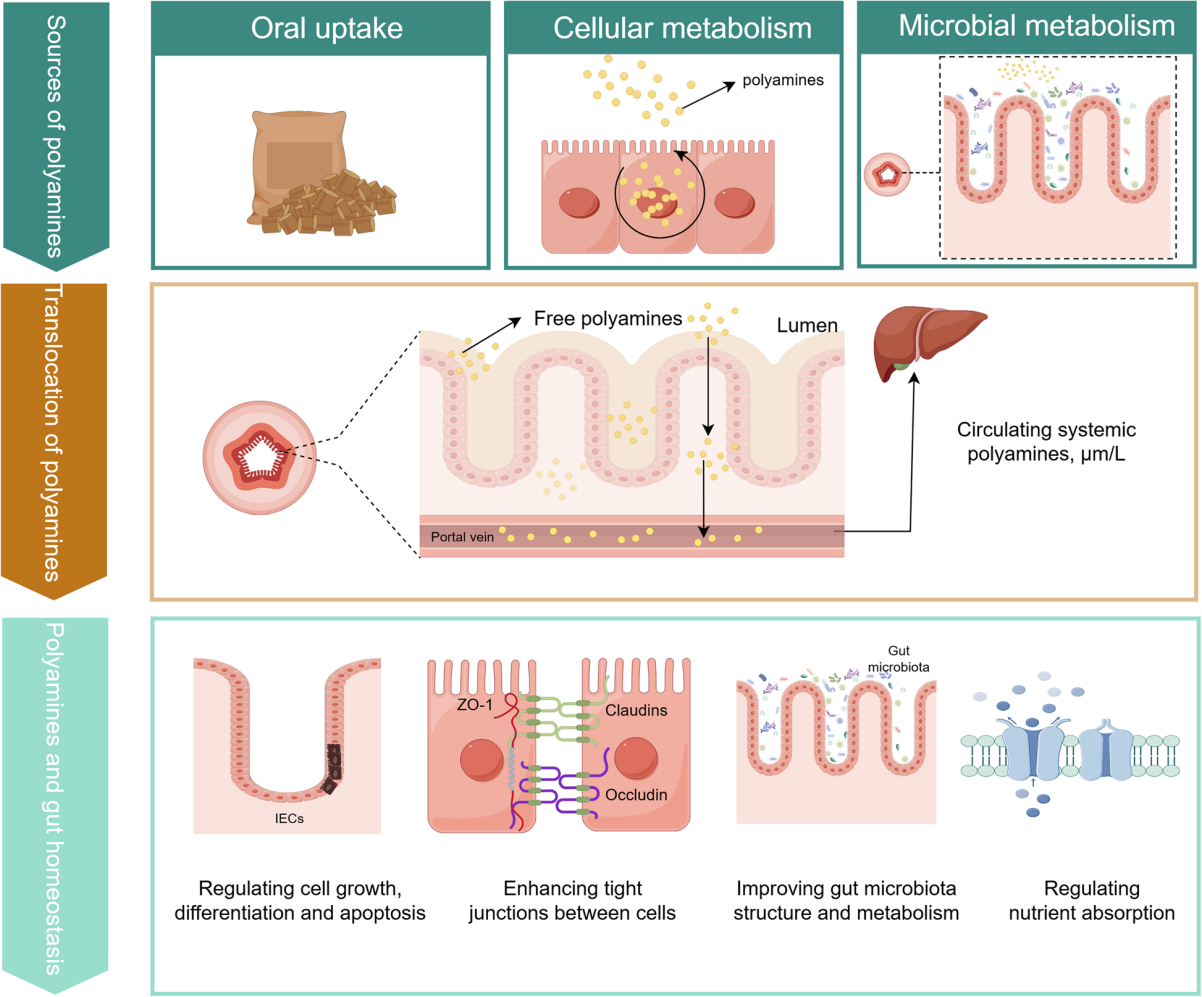

## Introduction

**Fig. 1:**
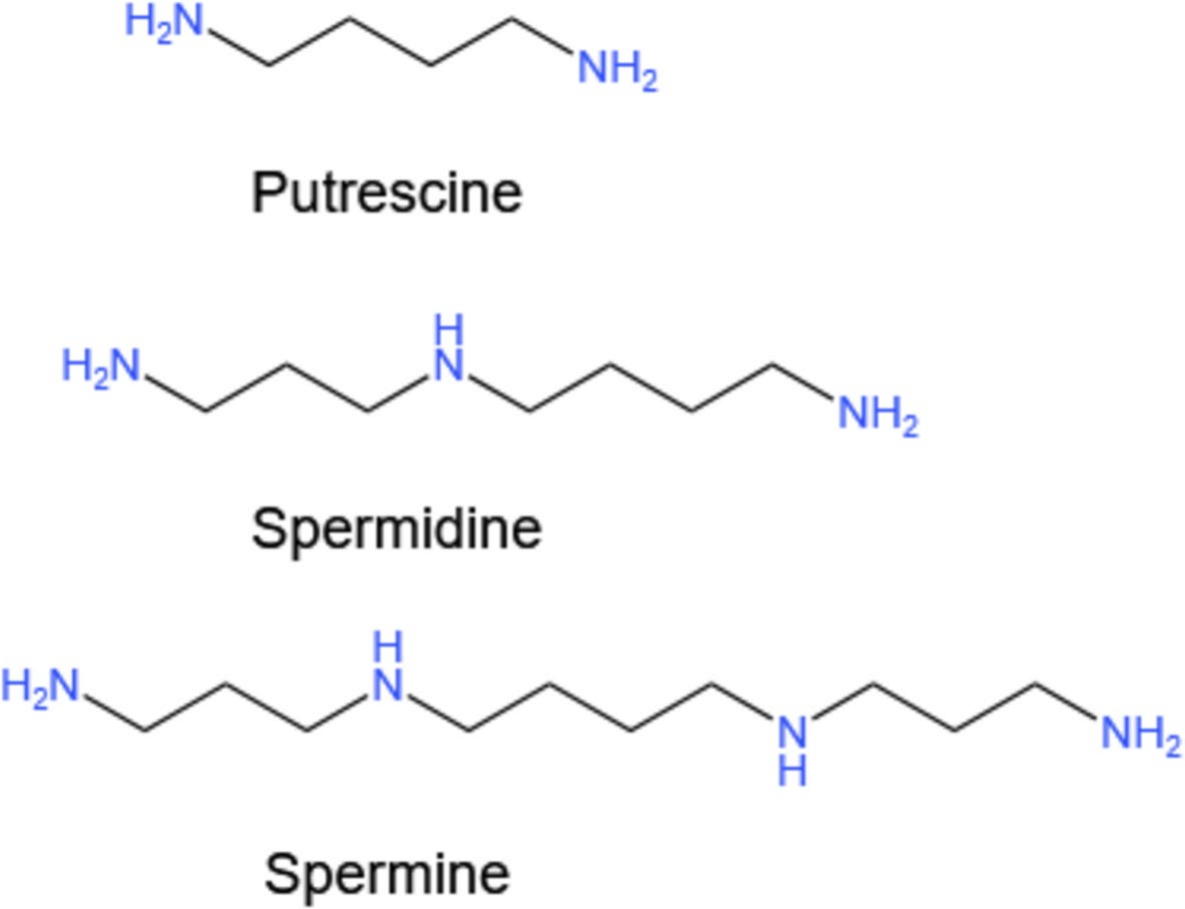
Putrescine, spermidine and spermine chemical structure

Polyamines, including putrescine and its downstream metabolites spermidine and spermine (Fig. 1), are widely present in microorganisms and higher plant and animal cells. They primarily exist in a bound state with anionic molecules such as DNA, RNA, specific proteins, ATP, and phospholipids. Polyamines regulate gene expression, maintain the stability of biological membranes, and promote the growth of cells and tissues [1–3]. Polyamines in animals usually originate from exogenous dietary supplementation, endogenous cellular metabolism and metabolic production by the intestinal microbiota; polyamines in the lumen of the upper part of the small intestine mainly originate from dietary intake, and putrescine and spermidine, which are metabolized by colonic microorganisms in the lower part of the intestinal tract, are considered to be important sources of polyamines [4, 5]. The intracellular polyamine pool is tightly regulated by various mechanisms, including the synthesis of amino acid precursors, cellular uptake mechanisms for acquiring polyamines from diet and gut microbiota, and degradation and excretion processes [6, 7]. Notably, higher levels of polyamines usually accompany rapidly proliferating and developing tissues, and polyamine levels vary in an age-dependent, tissue- and cell-type-specific manner with an overall decreasing trend during the normal life cycle [8–10]. The decrease of polyamine levels caused by aging can be remedied by feeding supplementation or regulation of intestinal microbial composition, thereby promoting colonic epithelial proliferation and macrophage differentiation, alleviating intestinal inflammation, and enhancing intestinal barrier. It can also be transferred to the blood through the intestinal epithelium to protect the kidney and liver or enhance the function of other tissues and organs of the host, such as through the induction of autophagy to enhance cognitive ability of the brain, and to improve the function of the heart [11–14]. However, when the polyamine metabolic pathway is impaired or abnormal, it is usually accompanied by an imbalance of homeostasis and the occurrence of diseases in the animal organism [15]. Therefore, the relative stability of polyamine levels not only plays an important role in maintaining the normal physiological activities of the organism, but also indirectly reflects the health status of the organism, but little is known about the specific mechanisms of polyamines in playing biological roles.

This review primarily summarizes recent advances in the study of polyamine metabolism and transport in gut microbiota and cells. It discusses potential metabolic pathways of spermidine in gut microbiota, providing new strategies to increase polyamine levels in the gut. Additionally, we consolidate information on the mechanisms through which polyamines regulate intestinal epithelial cells, alter gut microbiota composition, and maintain gut homeostasis. We also explore the connections between polyamines and nutrient absorption and metabolism.

## Source and metabolism of polyamines

### Polyamine biosynthesis

**Fig. 2:**
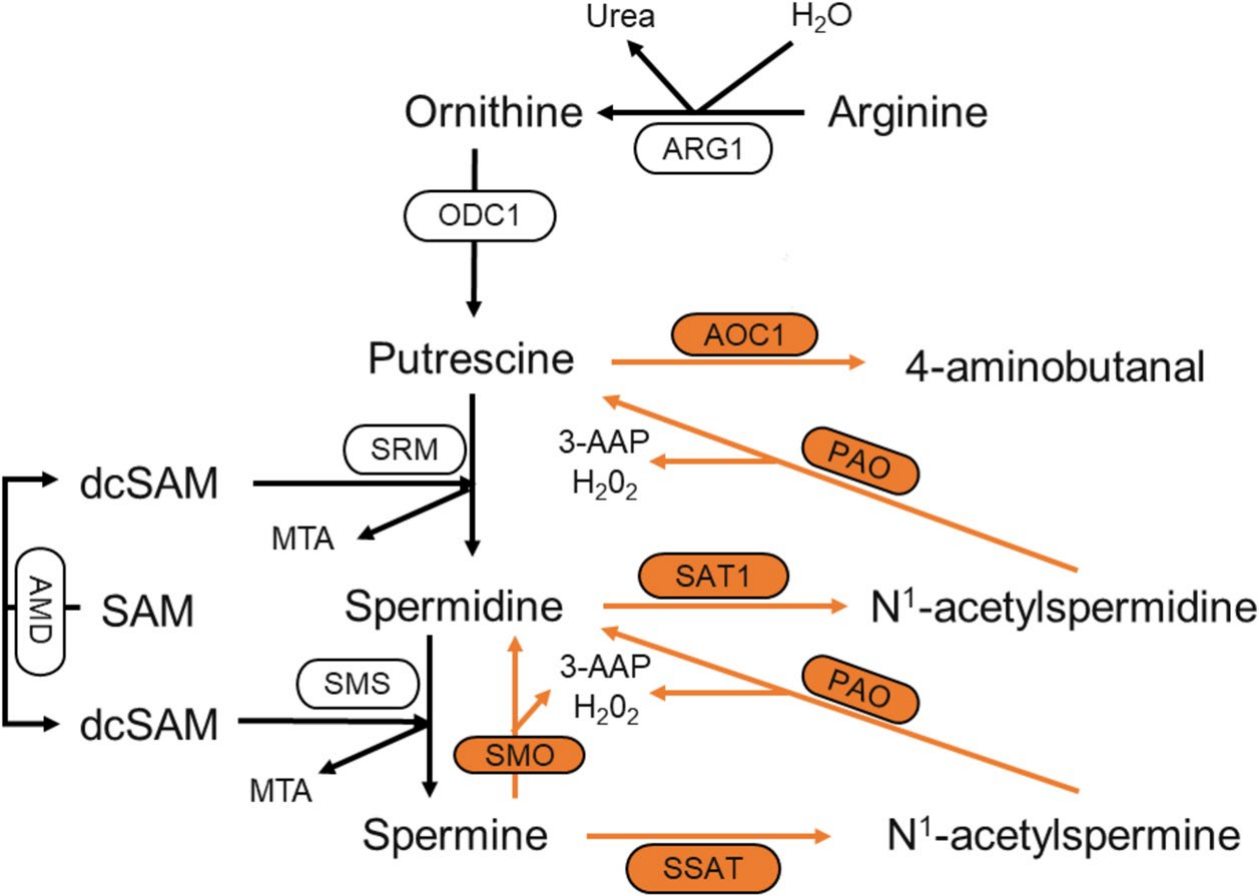
Polyamine synthesis and catabolism in mammals. The abbreviations used are as follows: ARG1, arginase 1; ODC1, ornithine decarboxylase; SAM, S-adenosylmethionine; AMD, S-adenosylmethionine decarboxylase; dcSAM, decarboxylated s-adenosylmethionine; SRM, spermidine synthase; MTA, methylthioadenosine; SMS, spermine synthase; SMO, spermine oxidase; 3-AAP, 3-acetylaminopropanal; SSAT/SAT1, spermidine/spermine-N^1^-acetyltransferase; PAO, polyamine oxidases; AOC1, amine oxidase copper-containing1; H_2_O_2_, hydrogen peroxide. Black is the anabolism pathway and orange is the catabolism pathway

Polyamines are present at certain levels in the animal gut and exhibit significant changes before and after feeding. Following ingestion, the concentration of polyamines in the intestinal lumen can rapidly reach several millimolar. Although the diet is the primary source of polyamines in the digestive tract, most of these compounds are absorbed in the upper sections of the digestive tract and are utilized for the growth and development of the organism [16]. The levels of putrescine and spermidine in the colon primarily depend on the gut microbiota, which metabolize precursor amino acids to release bioactive metabolites that regulate the host's overall balance [17]. In summary, besides dietary and host factors, the microbiota are also considered important determinants of polyamine levels in the digestive tract [18, 19]. The production of polyamines in the organism is a complex process. It involves amino acid precursors and intermediate metabolites. Polyamines can be synthesized from scratch by cells or metabolized by gut microbial catabolism. The process starts with the conversion of arginine to urea and L-ornithine by arginase 1 (ARG1). In mammals and fungi, putrescine is generated by ornithine decarboxylase 1 (ODC1), which relies on pyridoxal phosphate (PLP), a rate-limiting factor. The expression of ODC1 is induced in response to various stimuli. Its activity is regulated at the transcriptional, translational, and post-translational levels [20]. To ensure normal levels of polyamines, ODC1 is controlled by ubiquitin-independent proteasomal degradation mechanisms, mainly consisting of antizyme 1 (AZ1) and ODC antizyme inhibitor 1 (AZIN1), which interact with each other thereby releasing ODCs from the ODC-AZs complex and promoting the biosynthesis of spermidine [21]. S-Adenosylmethionine (SAM) produces decarboxylated S-adenosylmethionine (dcSAM) by the action of Adenosylmethionine decarboxylase 1 (AMD). The dcSAM is used as an aminopropyl donor for aminopropylation by spermidine synthase (SRM) and spermine synthase (SMS) to produce spermidine and spermine, and methylthioadenosine (MTA) [22, 23]. The detailed metabolic pathway is illustrated in Fig. 2. Arginine was found to be the major carbon donor for polyamine biosynthesis and glutamine was found to be a minor carbon donor supporting polyamine biosynthesis in T cells by ^13^C labeling of arginine, glutamine and proline. Therefore, arginine is now considered to be the major pathway for mammalian polyamine synthesis [24].

### Polyamine synthesis in microorganisms

**Fig. 3:**
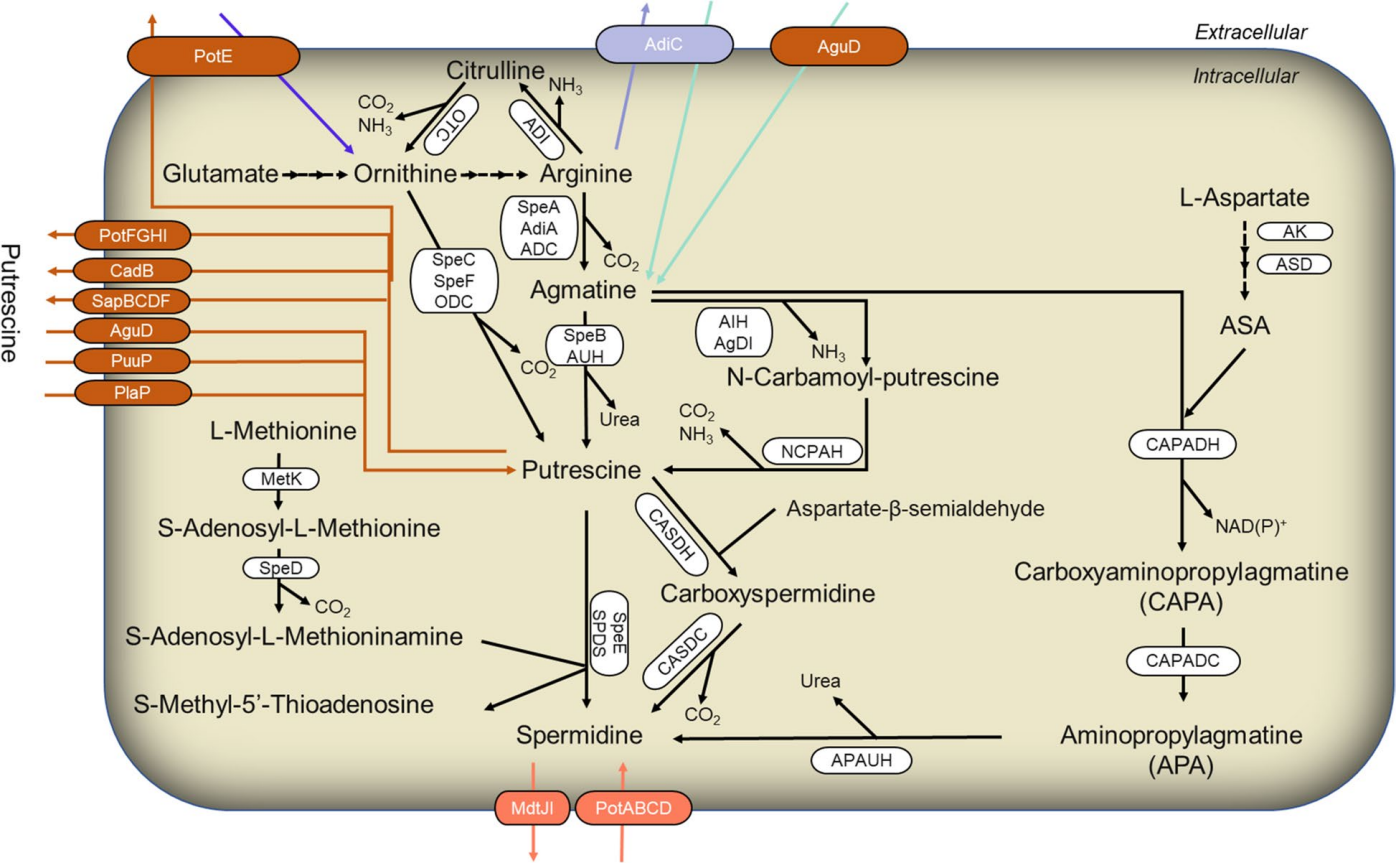
Polyamine biosynthetic and transport pathways in microorganisms. Polyamine biosynthesis and transport pathways previously described in microorganisms are integrated and elucidated. The abbreviations used are as follows: ADI, arginine desimidase; OTC, ornithine transcarbamylase; SpeA, AdiA, ADC, arginine decarboxylase; SpeB, AUH, agmatine ureohydrolase; SpeC, SpeF, ODC, ornithine decarboxylase; AIH, AgDI, agmatine deiminase; NCPAH, N-carbamoylputrescine amidohydrolase; CASDH, carboxyspermidine dehydrogenase; CASDC, carboxyspermidine decarboxylase; MetK, methionine adenosyltransferase; SpeD, S-adenosylmethionine decarboxylase; SpeE, SPDS, Spermidine synthase; AK, aspartokinase; ASD, aspartate-β-decarboxylase; ASA, aspartate-β-semialdehyde; CAPADH, carboxyaminopropylagmatine dehydrogenase; CAPA, carboxyaminopropylagmatine; CAPADC, carboxyaminopropylagmatine decarboxylase; APA, aminopropylagmatine; APAUH, aminopropylagmatine ureohydrolase. PotABCD, spermidine uptake protein; MdtJI, Spermidine output protein; AguD, PuuP, PlaP, Putrescine uptake protein; PotFGHI, CadB, SapBCDF, Putrescine output protein; PotE, Ornithine-putrescine transporter protein; AdiC, Arginine-agmatine transporter protein; AguD, Agmatine uptake protein

The microbiota in the gut, composed of bacteria, archaea, fungi, protozoa, and viruses, is considered the host's “digestive organ”. It is responsible for metabolizing undigested and unabsorbed nutrients in the gut, providing the host with essential nutrients such as vitamins, amino acids, short-chain fatty acids, and other bioactive compounds [25]. Similarly, polyamines can also be produced by gut microbiota. Unlike host polyamine metabolism, microbial polyamine production is a more complex process involving the uptake and export of intermediate products among different bacterial species [26]. Metabolomics studies of murine gut contents indicate that the concentrations of spermidine and putrescine in the intestinal lumen are dependent on the colonic microbiota, whereas spermine levels remain unaffected [18]. The 74 strains of enterobacteria that are associated with biogenic amine metabolism currently isolated from the human gut belong to several genera, including *Bifidobacterium*, *Clostridium*, *Enterococcus*, and *Lactobacillus*. These strains mainly utilize amino acid decarboxylases, either in a constitutively or inducible manner, to produce polyamines, with arginine decarboxylation being the main pathway for the production of putrescine [27, 28]. Both ornithine decarboxylase and arginine decarboxylase activities have been detected in bacteria and archaea. The expression and activity of these enzymes are regulated to adapt to the polyamine metabolic processes under various physiological conditions [29, 30]. In *Escherichia coli*, the constitutive *speC* and inducible *speF* are two ornithine decarboxylase genes that, along with the operon potE (ornithine-putrescine antiporter), are expressed under acidic conditions and high levels of ornithine [31]. These enzymes catalyze the decarboxylation of ornithine to produce putrescine. Arginine is first metabolized to agmatine by SpeA and SpeB and then converted to putrescine. The produced putrescine is further condensed with decarboxylated S-adenosylmethionine (AdoMet) under the action of SpeE to form spermidine [32]. In *Campylobacter jejuni*, the biosynthesis of spermidine relies on L-aspartate-β-semialdehyde (ASA). Putrescine is first converted to carboxyspermidine by carboxyspermidine dehydrogenase (CASDH) and then to spermidine by carboxyspermidine decarboxylase (CASDC) [33]. Many bacterial genomes encode homologs of CASDH and CASDC. For instance, among 56 abundant bacterial species within the genus *Bacteroides*, 20 species contain homologs of CASDC, which are crucial for spermidine biosynthesis. In bacteria and archaea, an arginine decarboxylase (ADC) pathway for polyamine biosynthesis is also present. For example, in *Campylobacter jejuni*, arginine is converted to agmatine by ADC. Agmatine is then hydrolyzed by agmatine ureohydrolase (AIH) to produce N-carbamoylputrescine [34]. In *Bacteroides thetaiotaomicron*, an important species within the gut microbiota, N-carbamoylputrescine aminohydrolase (NCPAH) converts N-carbamoylputrescine into putrescine [35]. The detailed metabolic process is illustrated in Fig. 3.

The gut microbiota is a complex mixed system, where the synthesis of metabolic products can be carried out either by single cells or through sequential reactions involving different bacterial species. In certain species such as *Enterococcus*, *Streptococcus*, *Clostridium*, and *Lactobacillus*, spermidine can be synthesized even in the absence of AdoMet decarboxylase (AdoMet DC) and SRM or their homologs. Therefore, polyamine metabolism and transport pathways are likely to span multiple bacterial species. Nakamura et al. [26] used isotope labeling to show that putrescine in the colon is produced by a collective biosynthetic pathway from different bacteria through complex metabolite exchange, thus proving this possibility. It was found that the concentration of putrescine in the supernatant of mixed culture of eight intestinal bacteria was higher than that of single bacterial culture, and a new putrescine synthesis pathway was found in the process of co-metabolism and transport of polyamines by *E. coli* and *Enterococcus faecalis* [36, 37]. When the pH decreases, the acid tolerance system of *E. coli* becomes active. The arginine-agmatine antiporter (Adi C) facilitates the uptake of environmental arginine, which is then converted to agmatine by arginine decarboxylase (Adi A) [38]. *E. faecalis* utilizes the agmatine-putrescine antiporter (Agu D) to take up agmatine produced by *E. coli*. Agmatine is hydrolyzed to N-carbamoylputrescine and ammonia by agmatine deiminase (Agu A), and subsequently converted to putrescine by N-carbamoylputrescine amidohydrolase (Agu B). This process not only generates ammonia but also produces ATP and CO_2_. Additionally, bifidobacteria, which produce acidic substances in the gut, can enhance this pathway of putrescine formation [39]. Jutta Noack et al. [40] fed indigestible polysaccharides such as pectin to rats, which produced large amounts of short-chain fatty acids and lowered intestinal pH, altered gut microbial composition and metabolism, and promoted the synthesis of polyamines by certain microorganisms such as *Bacteroides thetaiotaomicron* and *Fusobacterium varium*, confirming that polyamine formation in some bacterial species in the gut is stimulated by the provision of appropriate metabolic substrates.

Microbes play a crucial role in maintaining the homeostasis of polyamine metabolism, and studies on polyamine metabolism in microorganisms have been ongoing. Recently, a novel spermidine biosynthetic pathway known as the CAPA pathway was discovered in the model cyanobacterium *Synechocystis *sp*.* PCC 6803. Compared to the classical spermidine synthase-mediated pathway, the CAPA pathway requires less energy and does not involve putrescine. This pathway is widely distributed across 15 bacterial phyla, including Cyanobacteria, Proteobacteria, Firmicutes, and Bacteroidetes, providing new directions for the biosynthesis of polyamines and their derivatives [41]. However, some microbial polyamine anabolic pathways that have not been extensively studied remain unknown, and it is expected that new metabolic pathways will continue to be discovered in the future [32].

### Polyamine catabolism in microorganisms and their hosts

The overall levels of polyamines are regulated on one hand by biosynthesis and uptake mechanisms, and on the other hand by catabolism and efflux mechanisms [42]. Polyamine metabolism in mammalian cells occurs mainly by oxidative deamination in the presence of diamine oxidase and polyamine oxidase (PAO). Spermidine/spermine-N^1^-acetyltransferase (SSAT) is a key enzyme in the catabolism of polyamines present in the cytoplasm and is involved in catalyzing the first reaction in the polyamine degradation and export pathway. In the degradation pathway, acetyl-coenzyme A (CoA) serves as an acetyl donor. Spermidine and spermine are acetylated by SSAT, resulting in acetyl-spermidine and acetyl-spermine. These acetylated compounds can be excreted from the cytoplasm via transporter proteins in the cytoplasmic membrane, or they can be used as substrates for PAO. PAO oxidizes them to produce putrescine and spermidine by removing the acetamidopropanal group, which is then recycled back into the polyamine pool. Additionally, spermine can be directly oxidized to spermidine by spermine oxidase (SMO) [43, 44]. The whole catabolic process not only generating the corresponding polyamines but also produces potentially toxic by-products, such as H_2_O_2_ and aldehydes that cause damage to cells, DNA, etc. Consequently, oxidative damage may be exacerbated by increasing the level of H_2_O_2_ when polyamine catabolism is abnormal [45]. However, the effects of polyamines on organism health are not always beneficial. In pathological conditions, polyamine metabolic disorders may further accelerate the development of the disease. This dysregulation is common in cancer. The increase of polyamine biosynthesis and transport and the decrease of catabolism, resulting in elevated polyamine level, which in turn support the rapid proliferation of cancer cells. [15]. In Alzheimer's disease patients, higher concentrations of arginine and increased expression of the polyamine catabolic genes *SAT1* and *SMOX*, and decreased expression of the synthetic genes *SRM* and *ODC1* [46]. Similarly in Parkinson's patients, changes in polyamine levels also have been found, the decreased expression of polyamine metabolic enzyme SAT1 led to an increase in polyamine levels, which in turn reduced the cognitive performance of patients with Parkinson 's disease through the NMDAr pathway [47].

Due to the high concentration of polyamines causing damage to cells, the synthesis and metabolic processes synergistically maintain the dynamic stability of polyamines, which is no exception in microorganisms. They also prevent excessive accumulation of polyamines through the acetylation pathway. In *E. coli*, spermidine acetyltransferase (SAT), encoded by *speG*, catalyzes the acetylation and inactivation of spermidine. Silencing this gene leads to spermidine accumulation within the cell [48]. In *Bacillus subtilis*, the non-membrane-bound SSAT protein PaiA, a member of the N-acetyltransferase superfamily, acts as an N^1^-acetyltransferase for spermidine and spermine, preventing polyamine accumulation and cellular damage. In *E. faecalis* and *Staphylococcus aureus*, the SSAT homologs BltD and SpeG acetylate spermidine and spermine, with a preference for spermine [49, 50]. In *E. coli*, two pathways metabolize putrescine to succinate via the intermediate γ-aminobutyric acid (GABA). These are the Puu pathway and a pathway involving putrescine transaminase (YgjG) and γ-aminobutyraldehyde dehydrogenase (YdcW) [51–53]. In the Puu pathway, putrescine is transported into the cell by PuuP [54], and PuuA uses ATP to conjugate glutamate and putrescine, forming γ-glutamylputrescine [55]. This intermediate is oxidized by PuuB to γ-glutamyl-γ-aminobutyraldehyde, which is further oxidized by PuuC to γ-glutamyl-GABA. PuuD hydrolyzes the γ-glutamyl bond, producing GABA and glutamate [56]. GABA is deaminated by PuuE to form succinate semialdehyde, which is then oxidized to succinate by YneI [57]. Another pathway uses γ-aminobutyraldehyde as an intermediate, and putrescine is metabolized to GABA without undergoing γ-glutamylation [58]. Cultures of three strains of *Bifidobacterium* species (*B. breve*, *B. catenulatum*, *B. scardovii*) found that all of them could take up putrescine from the medium, but no putrescine was detected in the cells. This may be related to homologs of Gab D, Gab T, Pat A, and Pat D, which are involved in the transketolase pathway for putrescine degradation [59]. However, for some microorganisms, which lack both the proteome required for the transaminase pathway and do not possess the γ-glutamylation pathway, there may be an unknown pathway for polyamine degradation [60].

## Translocation of polyamines

Polyamine transport systems play a crucial role in determining polyamine homeostasis and distribution. Exogenous transport allows polyamines to enter the blood circulation through intestinal epithelial cells and transport to other tissues and organs to exert their biological functions. When radioactive-labeled polyamines were orally administered to rats, they were quickly absorbed but unevenly distributed across different tissues. Further studies revealed that polyamines preferentially accumulate in rapidly proliferating tissues [61]. Polyamines produced by gut microbiota are typically present in the colon lumen and the downstream small intestine. They enter the host organism through the colonic epithelial cells and are eventually transported to the proximal intestine via the portal vein circulation and the biliary system. This process is crucial for maintaining the polyamine pool and overall host health [13, 62]. The cellular uptake and efflux of polyamines are attributed to two different transporter systems and involve different carriers. The intensity of uptake is influenced by the cell's own polyamine demand. Cells with high proliferative activity and those experiencing polyamine depletion due to blocked polyamine synthesis pathways have a greater capacity for polyamine uptake [6]. Since the primary and secondary amino groups of polyamines are protonated outside the cell, they cannot cross the cell membrane through passive diffusion [63]. Therefore, there are different hypotheses regarding the transport of polyamines, including plasma transport, vesicle isolation, glycosaminoglycan-mediated endocytosis, and caveolin-mediated endocytosis [64]. Homology analysis of polyamine biosynthesis proteins has shown that some bacteria lack complete polyamine synthesis pathways and must import polyamines from their environment to support growth and adapt to environmental changes. For bacteria capable of synthesizing polyamines, uptake and efflux not only help maintain polyamine homeostasis but also offer convenience. Because polyamines are hydrophilic and positively charged, they cannot cross the hydrophobic cell membrane without specific transporters, making polyamine transport proteins essential for cells [41].

So far, five transport systems have been identified in *E. coli*. These include two polyamine uptake mechanisms, both belonging to the ATP-binding cassette (ABC) protein family. One system, which preference spermidine, consisting of PotA (ATPase), PotB and PotC (channel proteins), and PotD (substrate-binding protein). This system not only takes up spermidine but also has a low affinity for putrescine [65]. In *E. coli*, these four proteins are indispensable for the process of spermidine uptake [66]. The spermidine transporter encoded by *PotABCD* has also been found in *E. faecalis* and *Staphylococcus aureus*, compensating for their inability to synthesize polyamines, supporting normal cell growth and biofilm formation. Another system is specific for putrescine uptake and includes PotF (substrate-binding protein), PotG (ATPase), PotH and PotI (channel proteins) [67, 68]. In addition, the proton-dependent putrescine uptake protein PuuP and its homolog PlaP, which plays an important role when *E. coli* grows with putrescine as the sole carbon or nitrogen source. Unlike PotFGHI, PuuP is not inhibited by feedback inhibition from intracellular polyamines, but is inhibited by glucose [53, 54, 69]. There are also two polyamine-amino acid reverse transporter proteins: PotE (putrescine-ornithine) and CadB (putrescine-lysine). Both are proton-dependent putrescine uptake proteins that function in a neutral environment. SpeF converts ornithine and lysine to putrescine and cadaverine, respectively, by consuming a proton under acidic conditions. PotE exports putrescine through uptake of ornithine, while CadB outputs putrescine through uptake of lysine. This transport system is important for the acclimation of *E. coli* to acidic environment and for normal growth [70, 71]. Furthermore, the spermidine transporter protein (MdtJI) exports excess spermidine from *E. coli* cells, maintaining intracellular spermidine levels within a normal range [72]. Lastly, SapBCDF, another ABC transporter, exports putrescine from the cell [73]. A new polyamine transporter may exist during the collaboration of the two acid tolerance mechanisms in the co-culture of *E. faecalis* and *E. coli*. This collaboration induces putrescine synthesis, with *E. faecalis* utilizing the agmatine-putrescine transporter (AguD) to take up agmatine exocytosed by *E. coli*, and agmatine is converted to putrescine and then excreted extracellularly via AguD [36].

BLAST analysis revealed that proteins similar to PotD and PotF are distributed across several phyla, including Proteobacteria, Firmicutes, Actinobacteria, Fusobacteria, Cyanobacteria, Spirochaetes, Planctomycetes, Chlamydiae and Deinococci. Proteins with high similarity to PotE and CadB are found in Proteobacteria, Bacteroidetes, Firmicutes, and Actinobacteria, while those similar to PuuP and PlaP are limited to Proteobacteria, Firmicutes, Actinobacteria, Acidobacteria, and Sphingobacteria [74]. Apart from *E. coli*, new transporters have been identified in other bacteria. For instance, Blt is a spermidine exporter in *Bacillus subtilis* [75]. There are three PotD homologs NspS, PotD1 and PotD2 in *Vibrio cholerae*, among which NspS acts as a signal sensor to promote polyamine transport [76, 77], and four polyamine transport proteins DUR3, SAM3, GAP1 and AGP2 have been discovered in *Saccharomyces cerevisiae*, with DUR3 and SAM3 playing major roles in polyamine uptake. Additionally, five polyamine export transporters (TPO1-5) maintain intracellular polyamine stability [78, 79]. Cultivation of 13 human indigenous *Bifidobacteria* revealed that 10 species possess polyamine transport capabilities. BLAST analysis suggested the potential existence of novel polyamine biosynthesis and transport proteins [60]. Polyamines are endogenous active substances with multi-target regulation characteristics. Identification of these transporters can more comprehensively elucidate their transport processes and physiological mechanisms.

## Physiological functions and mechanisms of action of polyamines on microorganisms

### The role of polyamines in cell proliferation and differentiation

Putrescine and spermidine are common polyamines in bacteria. They are not only involved in core physiological processes such as gene expression and cell growth, but also affect iron carrier biosynthesis, endocytosed vesicle production, swarming motility, and formation of biofilms [80, 81]. Microbial reverse genetics studies have shown that gene deletion or polyamine depletion in polyamine metabolic pathways has a negative impact on cell survival and proliferation. Intracellular polyamines exist mainly in the form of polyamine-RNA complexes. For instance, in *E. coli*, 90% of spermidine is present in the cellular RNA complex, which maintains the RNA in a specific conformation and solubility, and is able to stabilise the RNA by interacting with other molecules (such as Mg^2+^). Polyamines can also bind to the outside of the DNA, allowing for intermolecular interactions to stabilise the double-stranded DNA [82]. Eukaryotic and archaeal use polyamines as aminobutyl donors, which are transferred to the ε-amino group of specific lysine residues of eIF5A under the action of deoxyhypusine synthase (DHS). The resulting deoxyhypusine lysine residues are then catalyzed by deoxyhypusine hydroxylase (DHH) to produce hypusine lysine [83]. All archaea encode DHS homologs, and inhibition of these homologs causes cell cycle arrest, indicating that polyamines or their precursors are essential for the normal growth of archaea. In eIF5A, lysine is modified by hydroxyputrescine, which activates eIF5A. This activation helps eIF5A bind to translationally active ribosomes, increasing the ratio of polyribosomes to monoribosomes. eIF5A plays a role in the elongation phase of translation. The level of polyamine content influences the degree of hydroxyputrescine modification of eIF5A, which in turn affects eIF5A function and cell proliferation [84–86]. Polyamine also regulate fungal cell differentiation. In some fungi, polyamines participate in spore germination, forming cell morphologies more resistant to adverse conditions than vegetative cells [87]. In conditionally pathogenic fungi, it was found that the heat-induced dimorphism conversion process of *Emergomyces africanus* and the conversion of *Candida albicans* between yeast state and hyphal state required the participation of polyamines [88, 89]. Additionally, spermidine is crucial for the normal growth of *Campylobacter jejuni* and *Pseudomonas aeruginosa* [34, 90], and polyamines are essential for the growth of *Bacillus subtilis* and *Streptococcus pneumoniae* [27, 91]. Putrescine is also indispensable for the growth of *Ralstonia solanacearum* [92].

### The role of polyamines in physiological stress responses

Physiological stress responses induced by oxidative stress, temperature changes, nitrosative stimuli, or other toxic compounds are present throughout the microbial life cycle. Polyamines provide resistance to both intracellular and environmental stresses. Intracellular polyamine levels changing in response to stress, and depletion of polyamines make cells more sensitive to stress. Reactive oxygen species (ROS) can cause DNA double-strand breaks and structural changes, damaging macromolecules within the cell. ROS levels increase as a byproduct of metabolism with increased metabolic rates. Superoxide dismutase (SOD) protects intracellular nucleic acids from damage caused by superoxide ions and other oxidants. Spermidine has been shown to be effective against alkyl, hydroxyl, and peroxy radicals, acting as a radical scavenger in conjunction with SOD to reduce oxidative damage [87]. In *Salmonella* Typhimurium, spermidine activates the stress response mechanism by regulating key antioxidant genes to counter ROS-mediated cytotoxicity and improve its survival in macrophages [93]. Polyamines also regulate the expression of stress response genes, mediating *E. coli* adapt to nitrative stress in the external environment. The *cadC* is an essential gene for nitrate stress tolerance in *E. coli*, its deletion leads to significantly reduced intracellular polyamine levels and increased sensitivity to acidified nitrite [94]. In fission yeast *Schizosaccharomyces* pombe, polyamine transporter Shp2 facilitates phosphate export in an Xpr1-independent manner and contributes to high phosphate tolerance [95]. The CadBA system, activated to counteract acidic stress, is regulated by the pH sensor CadC, which controls the expression of the *CadBA* operon. Although polyamines were not explicitly mentioned in this process, CadB functions as a lysine-cadaverine antiporter, so polyamines were likely involved in acid tolerance regulation [96, 97]. Engineered* Saccharomyces cerevisiae* strains with high spermidine levels exhibit resistance to chemicals like acetic acid and furfural, which inhibit microbial growth, metabolism, and ethanol fermentation [98]. Moreover, the survival of gut microbiota under acidic conditions depends on polyamines. In *E. coli*, polyamines reduce cAMP levels, thereby regulating the expression of the *gadA* and *gadB*, which influence the bacterium's sensitivity to acidic environments [99].

### The impact of polyamines on microbial biofilms

Polyamines are closely related to the motility and attachment of bacteria, the formation and function of biofilm [100]. Biofilms are aggregates of bacteria or other microbial communities in the extracellular matrix, including polysaccharides, proteins, extracellular nucleic acids and lipids. These membranes can form on living and non-living surfaces in response to specific environmental stimuli, thereby protecting the cell from the extracellular environment [101]. The permeability of the outer membrane of Gram-negative bacteria to hydrophilic compounds mainly depends on porins, which are homotrimeric transmembrane proteins. In *E. coli*, the porins OmpC and OmpF interact with polyamines in a voltage-dependent manner. Putrescine and spermidine bind to aspartate residues on OmpC and OmpF, altering their charge and pore size, leading to channel closure and reduced outer membrane permeability. Therefore, polyamines regulate bacterial outer membrane permeability [102, 103]. Disruption of the spermidine synthase gene (*speE*) in *E. coli* results in severe biofilm formation defects, which is restored by supplementation with spermidine, and biofilm formation is further enhanced by intracellular accumulation of spermidine [104], the absence of *potABCD* operon also impaired the biofilm formation of *Streptococcus pneumoniae*, and the addition of more polyamines to the medium stimulated the biofilm formation [105]. In *Yersinia pestis*, biofilm formation is positively correlated with exogenous putrescine levels but not with spermidine, possibly due to polyamine uptake proteins; putrescine exerts its effects after entering the cell and being converted [106]. Similarly, spermidine promotes biofilm formation in *Bacillus subtilis* by regulating the transcription of the extracellular polysaccharide and *TasA* operons through the factor *slrR* [107]. However, in *Vibrio cholerae*, spermidine uptake and extracellular spermidine inhibit biofilm formation through the NspS/MbaA signaling pathway [76]. Finally, putrescine mediates biofilm matrix degradation in *Shewanella oneidensis*, and blocking the putrescine biosynthetic pathway enhances biofilm cohesiveness and performance [108].

### The impact of polyamines on gut microbiota

Polyamines are not only metabolically produced by gut microbes but also influence microbial growth and colonization. The animal digestive tract hosts a large microbial community, where polyamines interact with the gut flora to maintain host health and improve metabolic phenotypes in the gut. There are two possible mechanisms for the effect of polyamines on the intestinal microbiota. On the one hand, polyamines serve as growth factors and preferred substrates for gut microbes. On the other hand, polyamines act as bioactive modulators, regulating probiotic colonization patterns and inhibiting harmful bacterial growth. Additionally, polyamines can also change the pH by increasing the volatile fatty acids in the intestine [109]. These findings provide new insights into the regulation of intestinal microbial growth and colonization by polyamines. Feeding neonatal mice with polyamine-rich infant formula showed higher levels of *Bifidobacterium*, *Akkermansia-*like bacteria, and *Lactobacillus-Enterococcus* group, promoting a healthy mucosal status, confirming that polyamines can modulate microbial colonization patterns in the gut [110]. *Alistipes* and *Turicibacter* are considered pathogenic bacteria associated with colitis, and spermidine supplementation significantly reduced their abundance in the gut of mice while increasing *Lactobacillus* levels. However, it also decreased beneficial bacteria such as *Odoribacter* and *Romboutsia* [111]. *Bacteroides* and *Parabacteroides* increased, while *Prevotella* and *Desulfovibrionaceae* decreased in the intestine of abdominal aortic aneurysm (AAA) mouse model. Exogenous supplementation of spermidine alleviated the imbalance of intestinal flora and helped prevent the development of AAA [112]. After piglets were fed a diet containing spermine, the number of *Lactobacilli*, *Bifidobacteria* and total bacteria in colon and cecal chyme increased, while the number of *E. coli* decreased [113]. Similarly, polyamines improve gut microbiota structure and health in poultry [114]. Although many studies suggest that polyamines influence the composition and diversity of gut microbiota (Table 1), the interaction between the two remains unclear.

**Table 1:** Effects of exogenous polyamines on gut and gut microbiota in different animal models

Polyamine	Animal model	Effects on intestine	Effects on gut microbiota	Reference
Putrescine	Weanling piglets	Promoting intestinal development and improves immune function	Increased *Lactobacillus* and *Bifidobacterium*; decreased* E. coli*	[115]
	Weanling piglets	Mitigating intestinal atrophy via anti-inflammatory effects		[116]
	*Cyanopica cyanus*	Reducing faecal IL-6, improving nutrient absorption, alleviating metabolic stress	Reduced *Clostridium perfringens*, increases lactic acid bacteria and enterobacteria, and benefits *C. cyanus* colonization	[117]
Spermidine	Mice	Promoting jejunum and ileum villus growth	Increased lactic acid bacteria; reduced *Turicibacter* and *Alistipes*, affected the balance of intestinal flora	[111]
	Mice	Suppressing inflammatory bowel disease via anti-inflammatory macrophages and epithelial barrier maintenance	Prevented *Firmicutes/Bacteroidetes*-to-*Proteobacteria* shift; maintained microbiota homeostasis	[118]
	Mice	Improving colon inflammation and delaying tumor development	Counteracting disease-induced *Prevotella* reduction; increasing *Proteobacteria* and *Deferribacteres*	[119]
	Mice	Rescuing intestinal barrier defects via AhR-Nrf2 and AhR-STAT3 pathways		[120]
Spermine	Piglets	Promoting ileal health via enhanced antioxidant capacity, barrier function, and metabolic regulation	Increased *Lactobacillus* spp., *Bifidobacterium* spp., and total bacteria; decreased *E. coli* in cecal/colonic digesta	[113]
	Suckling piglets	Accelerating intestinal maturation and enhancing antioxidant status		[121]
	Suckling rats	Accelerating gut development and enhancing antioxidant capacity through prolonged supplementation		[122]
	Weaning rats	Regulating intestinal development and enhancing jejunal antioxidant status		[123]

## Polyamines and intestinal homeostasis

### Polyamines regulate the proliferation of intestinal epithelial cell layers

The intestinal barrier system consists of the mucus layer, intestinal epithelial cells (IECs), tight junctions (TJs), immune cells, and gut microbiota. The intestinal epithelium forms the largest mucosal surface in the body, composed of a single layer of cells, including crypts and villi [124]. It forms a crucial barrier to protect the body from pathogenic microorganisms, viruses, and various harmful substances [125]. Among these components, IECs are important players in intestinal homeostasis, and multifunctional intestinal epithelial stem cells located in the crypts are constantly proliferating and replicating [126]. The integrity and efficacy of the intestinal barrier depends on the dynamic balance between apoptosis, proliferation, migration, differentiation, and inter-cellular interactions of IECs [127]. The supply of polyamines to dividing cells in the crypt is essential for normal intestinal epithelial renewal and repair of damaged mucosa [128, 129]. Polyamine levels are also biomarkers in the proliferation process of intestinal mucosa, with high levels of spermine and spermidine enhancing intestinal immune barrier function by reducing intestinal mucosal permeability [130]. When cells are stimulated to grow and divide, intracellular polyamine levels rapidly increase. Inhibiting ODC activity reduces polyamine levels in tissues, inducing intestinal mucosal atrophy and hindering intestinal epithelial renewal [131]. Oral administration of polyamines promotes intestinal epithelial cell proliferation and enhances mucosal repair after injury [132].

The regulation of polyamines is a central convergence point for multiple signaling pathways that drive various epithelial cell functions, controlling the expression of genes related to proliferation, arrest, and apoptosis. This includes the regulation of numerous growth-promoting proteins such as c-Myc, c-Fos, and c-Jun, as well as growth-inhibiting factors like p35, nucleophosmin (NPM), JunD, TGF-β, TGF-β receptors, and Smads [133–137]. When the polyamine level in the cells increases, the expression of growth-promoting genes is increased by activating the transcription of genes, while the expression of growth-inhibiting genes is inhibited. ODC overexpression increases the polyamine levels in IECs, stimulates the expression of growth-promoting protein genes, and accelerates the transition from the G1 to the S phase of the cell cycle, contributing to the stimulation of IECs proliferation. In contrast, a decrease in polyamine increases the level of inhibitory factors, leading to growth arrest [138]. In rat intestinal mucosal injury models, the stimulation of mucosal injury repair is accompanied by increased polyamine levels and enhanced expression of *c-Myc*, *c-Fos*, and *c-Jun* genes. By inhibiting ODC with DFMO to reduce polyamine levels, the expression levels of these genes also decrease, delaying the healing of damaged intestinal mucosa [139, 140]. As polyamine levels decrease, the levels of growth-inhibitory factors such as TGF-β and Smads in intestinal epithelium significantly increase [141]. In addition, polyamines can regulate the apoptosis of IECs through Akt kinase, ATF-2, XIAP and NF-κB signaling pathways [142–145].

Polyamines are not only involved in the process of gene transcription, but also influence the transport, stability, and translation of post-transcriptional mRNA. RNA-binding proteins (RBPs) and miRNAs are crucial in this process. AU-rich element binding proteins (ARE-binding proteins, AUBPs) are significant members of the RBP family. They bind to AREs in the 3'untranslated region (3'UTR) of various mRNAs and influence their stability. They also regulate translation and mRNA export, controlling the expression of multiple important proteins involved in cellular functions [146, 147]. For instance, AUBPs regulate the mRNAs of various inflammatory cytokines to modulate the inflammatory response, these AUBPs include AU-rich element-RNA binding factor 1 (AUF1) and Human antigen R (HuR) [148]. *JunD* mRNA is the common target of HuR and AUF1, and there is a competitive relationship between them. The decrease of polyamine level in cells can enhance the binding of HuR to *JunD* mRNA and reduce the transcription level of JunD related to AUF1, thus stabilizing *JunD* mRNA. Conversely, increased cellular polyamines inhibited the interaction of *JunD* mRNA with HuR and enhanced its binding to AUF1, resulting in the inhibition of *JunD* expression [149]. HuR can regulate the stability and translation of multiple mRNAs within cells [150], thus HuR is generally considered a post-transcriptional enhancer of biological processes in intestinal epithelial homeostasis. Knocking out *HuR* in mouse intestinal epithelial tissue inhibits epithelial cell renewal and delays mucosal repair following injury [151]. Although reduced polyamine levels do not alter the total amount of HuR in cells, they affect its transport between the nucleus and cytoplasm, leading to increased accumulation of HuR in the cytoplasm [135]. Adenosine 5'-monophosphate-activated protein kinase (AMPK) is a protein kinase that regulates metabolism and energy stability. Polyamine-mediated AMPK activation in IECs can regulate the phosphorylation and acetylation of Impα1 to regulate the subcellular localization of HuR. The decrease of polyamine content leads to the inactivation of AMPK-involved Impα1 pathway and the accumulation of HuR in the cytoplasm [152]. MEK-1 is a signal transduction enzyme that plays a role in cell function. Polyamines in IECs reduce the stability of *MEK-1* mRNA and inhibit its translation process by inhibiting the binding of HuR to MEK-1 transcripts [153]. Furthermore, studies have shown that polyamines can regulate the translation of *c-Myc* in IECs through Chk2-mediated phosphorylation of HuR [154].

### Polyamines regulate connections between intestinal epithelial cells

Differentiated IECs forms a whole through protein complex connection, establishes a selective permeability barrier, prevents the random diffusion of substances and maintains its own polarity. It is an important part of the intestinal mechanical barrier, and ultimately maintains the integrity of intestinal epithelial cells and reduces the occurrence of intestinal diseases [155]. The connection between IECs include TJs, adhesive junctions (AJs) and desmosomes. Among them, TJs are the most critical type of connection between intestinal cells. TJ proteins are essential for influencing IECs function and determining the defensive capability of the intestinal mucosal barrier, including transmembrane protein Claudin, Occludin and cytoplasmic scaffold protein Zonula Occludens (ZO) family [156, 157]. Research has shown that polyamines regulate the synthesis and stability of these junction proteins. Inhibiting polyamine synthesis by DFMO decreases Occludin protein levels, but does not affect its mRNA expression. The contents of ZO-1, ZO-2, Claudin-2 and Claudin-3 decrease in polyamine-deficient cells [158]. Another study indicated that HuR binds to the 3'UTR of occludin mRNA, enhancing its translation. However, this binding depends on Chk2-dependent phosphorylation of HuR. As polyamine levels decrease, so does Chk2, thereby affecting the translation of occludin mRNA [159]. Below the TJs are AJs, which are rich in cadherins and mediate strong intercellular adhesion, playing a significant role in the formation and regulation of the epithelial barrier. Polyamines regulate E-cadherin expression at the transcriptional level through Ca^2+^ and the transcription factor *c-Myc*, promoting the function of the intestinal epithelial barrier and helps maintain the integrity of the intestinal mucosa [160, 161]. Connexin 43 (Cx43), a gap junction protein, is essential for intercellular communication and nutrient diffusion. HuR directly binds to *Cx43* mRNA through its 3'UTR in the IECs, stabilizing *Cx43* mRNA and enhancing its translation. This promotes the function of Cx43-mediated gap junctions, which play a key role in regulating intestinal epithelial barriers [162]. In IECs, polyamines alter the ratio of stromal interaction molecule 1 to stromal interaction molecule 2 (STIM1/2), controlling transient receptor potential channel 1 (TRPC1)-mediated Ca^2+^ signaling and influencing cell migration for epithelial remodeling after injury [163]. Additionally, intracellular Ca^2+^ concentration and membrane potential are affected by the expression of the voltage-gated potassium channel (Kv1.1), which is regulated by polyamines [164]. Wild-type polyamine-producing *E. coli* and mutant strains missing the polyamine synthesis gene were colonized in the mouse intestine, and polyamines produced by *E. coli* promoted the proliferation of colonic epithelial cells and macrophages, and reduce the risk of colitis in mice [14]. Given the significant role of polyamines in maintaining intestinal homeostasis, they also show potential in promoting the growth and development of gastrointestinal and colonic mucosa in newborn mammals, thereby reducing intestinal diseases [165].

### Polyamines enhance intestinal antioxidant damage

The stability of the intestine also depends on the antioxidant capacity and autophagy induction of polyamines. Polyamines improve the antioxidant defense of the intestine by increasing free radical scavenging capacity and enzymatic and non-enzymatic antioxidant capacity, and alleviate the intestinal oxidative damage caused by weaning stress [121]. Glutathione-S-transferase (GST) is a detoxifying enzyme that protects cells by clearing toxic substances. Glutathione (GSH) is a non-enzymatic antioxidant that can conjugate with H_2_O_2_ and lipid hydroperoxides, while one of the primary functions of GST is to promote the reaction between GSH and various endogenous and exogenous electrophilic compounds, producing less toxic or non-toxic substances. Exogenous spermidine intake can increase GST activity and elevate GSH levels in the intestine, mitigating oxidative damage [113]. In an acute colitis mouse model induced by dextran sulfate sodium (DSS), spermidine reduces intestinal inflammation by promoting anti-inflammatory macrophages, maintaining a healthy microbiome, and preserving epithelial barrier integrity in a protein tyrosine phosphatase non-receptor type 2 (PTPN2)-dependent manner [118]. Another study showed that the occurrence of colitis was accompanied by a decrease in SMO content in tissues and was negatively correlated with the severity of the disease. After treatment with spermidine, the symptoms of colitis were significantly improved and the development of tumors was inhibited [119]. In conclusion, current research has revealed that polyamines maintain intestinal homeostasis through various mechanisms, ensuring intestinal health and aiding in the efficient digestion and absorption of nutrients.

## Polyamines regulation of nutrient absorption and metabolism

The unique physiological functions and widespread presence of polyamines determine their crucial role in regulating glucose, lipid, and energy homeostasis. Moreover, the acetylation reactions involved in polyamine catabolism require CoA consumption, linking polyamine catabolism with lipid oxidation, energy expenditure, and glucose metabolism. Polyamines have been shown to have an effect on the regulation of metabolic disorders and energy homeostasis in different mouse models [166, 167]. In addition, they are involved in metabolic processes in rat blood, including cell membrane metabolism, lipid metabolism, glucose metabolism, and amino acid metabolism [168].

Polyamines exert significant physiological effects on lipid metabolism, particularly in the differentiation of preadipocytes into adipocytes. The 3T3-L1 cell line is a widely used model for studying adipose metabolism, and spermidine is essential for adipogenic differentiation in 3T3-L1 fibroblasts [169]. Spermidine regulates the fat metabolism process by upregulating fibroblast growth factor 21 (FGF21) signaling and its downstream PI3K/AKT and AMPK pathways [170]. Another study found that exogenous putrescine promoted adipogenic differentiation, increased intracellular lipid accumulation and the expression of adipogenic genes in 3T3-L1 preadipocytes [171]. In contrast, supplementing with spermine inhibited the expression of CCAAT enhancer binding protein α (*C/EBPα*) mRNA, affecting the adipocyte differentiation process and inhibiting lipid accumulation [172]. Furthermore, spermine regulated the differentiation of adipose tissue-derived mesenchymal stem cells into osteoblasts while inhibiting adipogenesis [173]. Since natural polyamines can interconvert intracellularly, altering the intracellular ratio of spermidine to spermine also affects adipocyte differentiation [174]. When polyamines are depleted in cells, the structure and function of mitochondria are greatly affected, and the decrease of mitochondrial fatty acid oxidation leads to lipid accumulation [175]. Oral administration of spermine to mice fed with high-fat diet found to alleviate the increase in body weight, blood triglycerides and visceral fat caused by high-fat diet [172]. Interestingly, oral administration of spermidine in obese mice also showed a similar effect. The average diameter of visceral fat and adipocytes decreased, and it had higher glucose tolerance and insulin sensitivity. Unlike spermine, spermidine, as an autophagy inducer, plays an important role in alleviating weight gain caused by high-fat diet [176]. Additionally, adding spermidine in an atherosclerotic mouse model inhibited lipid accumulation and necrotic core formation by stimulating cholesterol efflux [177]. Studies on the mechanisms by which polyamines regulate lipid metabolism have shown that the activity of key enzymes in the polyamine metabolic pathway, such as SSAT, ODC, SRM, and SMS, also affects lipid metabolism. Polyamines regulate lipid metabolism through involvement in pathways such as AMPK, mammalian target of rapamycin (mTOR), and C/EBP [178–182].

There is a close relationship between lipid metabolism and glucose metabolism. Reports on the regulation of glucose by polyamines can be traced back to the study of brush border membrane vesicles ( BBMV) at the end of the 20^th^ century. Intestinal luminal polyamines affect the number of glucose carriers in the cell membrane and also increase the affinity of carriers for glucose through direct interactions with vesicular membranes, thereby facilitating glucose uptake and determining the maximal rate of glucose transport [183]. Further investigation has revealed that polyamines enhance glucose absorption in the small intestine by rapidly increasing the glucose transporter protein SGLT1 on the brush border membrane [184]. Oral administration of polyamines to obese mice has shown that spermidine intake is negatively correlated with obesity, significantly improving diet-induced insulin resistance, enhancing glucose utilization, and effectively ameliorating obesity and glycemic status [185, 186]. In the rat small intestine putrescine can also be converted to succinic acid as a source of energy [187]. In addition, polyamines are involved in pancreatic development and regulation of β-cell function, and in human serum spermidine levels are negatively correlated with the triglyceride-glucose index [188, 189]. In polyamine depletion-mediated Warburg-like effects, cells shift from aerobic respiration to glycolysis, leading to an alteration in metabolic pathways. In summary, polyamines play a critical role in maintaining normal energy demands and vital activities by regulating mitochondrial function, glucose and lipid metabolism, and cellular energetics [175].

## The impact of polyamines as health modulators on animal production performance and their application prospects

Polyamines, a class of endogenous bioactive molecules with multi-target regulatory properties, play indispensable roles in maintaining cellular homeostasis in mammals. Depletion of polyamines has been shown to disrupt normal cell growth and proliferation. Substantial experimental evidence demonstrates that exogenous polyamine supplementation confers multiple physiological benefits, including improved cardiovascular health, enhanced neuroprotection, immune regulation, tumor suppression, mitigation of inflammatory damage, and increased stress tolerance, while exhibiting potential to delay organismal aging and extend lifespan [9, 190]. Notably, spermidine has been ranked among the top eight most promising anti-aging substances due to its biological efficacy [191].

**Fig. 4:**
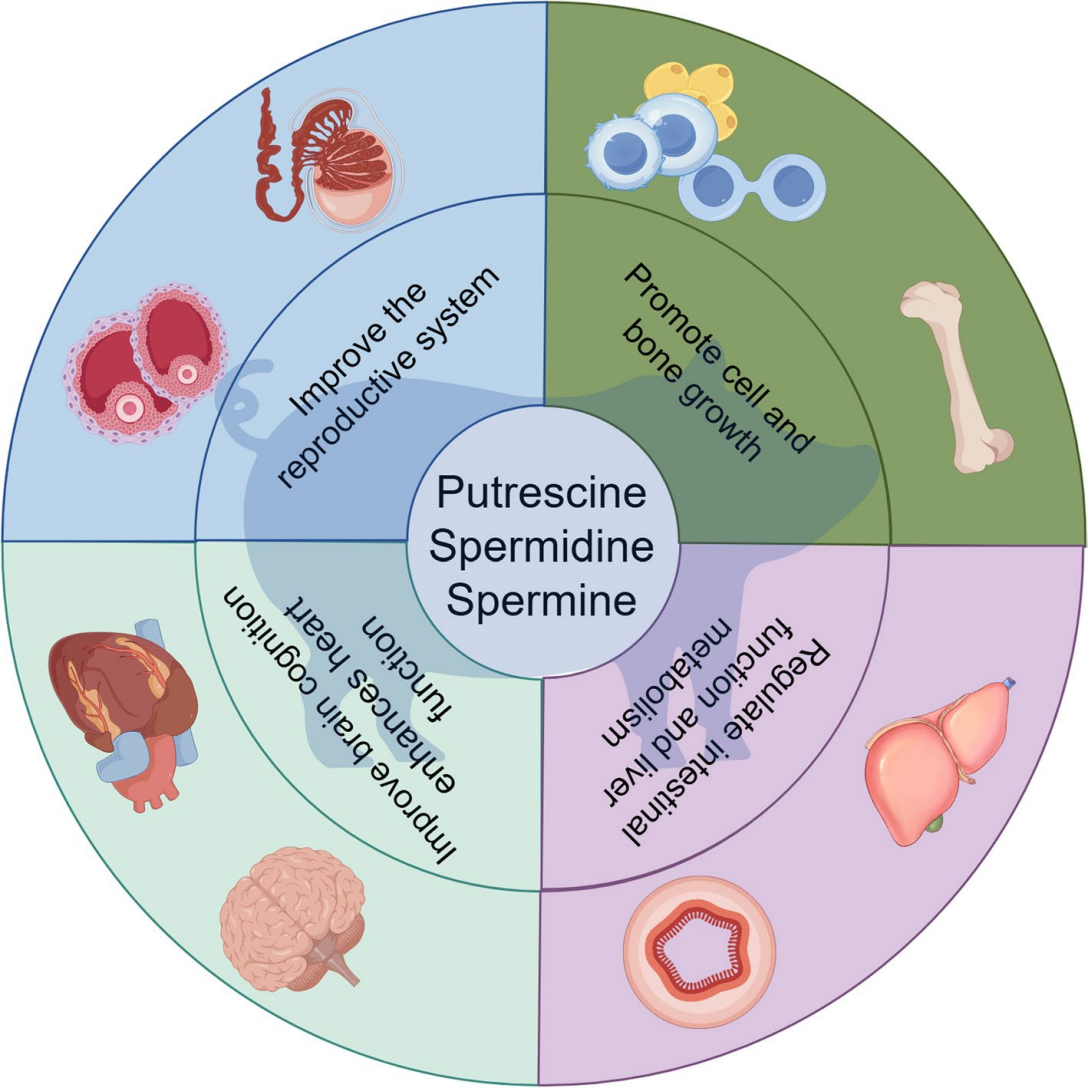
Effects of polyamines on mammalian health

As the most abundant polyamine in mammals, spermidine promotes ovarian follicular development and oocyte maturation in aged individuals through autophagy activation and mitochondrial function enhancement, thereby improving fertility and litter size [192–194]. Furthermore, spermidine and spermine alleviate lipopolysaccharide (LPS)-induced mitochondrial dysfunction and apoptosis in sperm via a CK2-dependent mechanism, significantly improving sperm quality and prolonging the preservation efficiency of porcine semen [195]. In ruminants, polyamines are critical for establishment and maintenance of pregnancy during the peri-implantation period of gestation [196]. These findings suggest that polyamines may serve as novel regulators for reproductive health optimization. Polyamines also enhance intestinal barrier integrity by upregulating tight junction proteins, reducing gut permeability and mucosal atrophy in piglets. Their capacity to stimulate intestinal stem cell proliferation significantly increases the villus height/crypt depth ratio, which correlates with decreased diarrhea incidence and enhanced immune function [115, 116]. From a systems biology perspective, dietary polyamine intake activates systemic cellular metabolic reprogramming pathways, exerting anti-aging effects [197] while improving nutrient absorption and whole-body metabolic efficiency (Fig. 4) [198, 199].

Although polyamines have demonstrated significant biological benefits in foundational studies, their application in livestock production remains underexplored. Future translational opportunities may involve their development as functional feed additives or therapeutic agents to enhance overall health. However, critical challenges must be addressed, including elucidation of dose-response relationships and mechanistic pathways governing polyamine-mediated health regulation.

## Conclusion

In this review, we summarize the complex and diverse functions of polyamines in cells. Current evidence indicates that polyamines promote anti-aging processes, enhance stress adaptation, mitigate intestinal damage, and exhibit dual roles in both anti-inflammatory and antioxidant responses. While polyamines negative effects on pathologically relevant diseases such as cancer, Alzheimer's disease, and Parkinson's disease. We don't discuss much about the detrimental effects of polyamines in this article. Dietary intake remains the main source of polyamines and cellular polyamine pools are tightly regulated by synthesis, degradation, uptake and efflux. Notably, gut microbiota emerge as key contributors to maintaining intestinal spermidine levels. Therefore, our discussion has focused on polyamine metabolic pathways and their interplay between microbial communities and the host. However, key microbial components governing polyamine dynamics—including transporters for uptake/efflux, utilization pathways, and biosynthetic modules—remain poorly characterized. Specifically, systematic identification of genes regulating microbial polyamine metabolism and high-throughput screening for high-efficiency probiotic strains capable of polyamine synthesis represent critical research priorities. Further elucidating of how living cells maintain polyamine homeostasis through precise regulation of their biosynthesis, interconversion, catabolism, and conjugation is essential to achieve optimal bioactive concentrations. Such mechanistic insights are critical for understanding how polyamines modulate specific biological processes under both physiological and stress conditions, thereby advancing our comprehensive knowledge of their multifunctional roles across diverse biological systems.

Intestinal health is crucial for the organism and is the basis for the digestion and absorption of nutrients and for ensuring normal life activities of the organism, and its homeostasis is maintained through a variety of mechanisms. Polyamines have a wide range of cellular functions in the intestinal epithelium and are involved in a variety of physiological and pathological processes. Here we emphasize the role of polyamines as active small molecules in maintaining the integrity of the intestinal epithelium as well as in the regulation of lipid, amino acid and glucose metabolism. The growth of the intestinal mucosa is dependent on the efficient supply of polyamines to dividing cells in the crypts. There is growing evidence that polyamines are involved in cell growth, differentiation and apoptosis by regulating gene expression, and show great potential in attenuating oxidative damage, increasing antioxidant status and digestive enzyme activity, and regulating intestinal flora. Although exogenous polyamines are effectively alleviate intestinal inflammation and restore barrier function, their clinical translation remains challenging. With the rapid development of polyamine biology, polyamine metabolism and transport have been used as important targets for the treatment of intestinal disorders and the improvement of body health. However, polyamines are widely used as functional substances, and a lot of work still needs to be done in the future.

## Data Availability

Not applicable.

## Abbreviations

ADC: Arginine decarboxylase
Adi A: Acid-induced arginine decarboxylase
Adi C: Arginine-agmatine antiporter
AdoMet: S-adenosylmethionine
AdoMet DC: AdoMet decarboxylase
ARG1: Arginase 1
Agu A: Agmatine deiminase
Agu B: N-carbamoylputrescine amidohydrolase
Agu D: Agmatine-putrescine transporter
AIH: Agmatine ureohydrolase
AJs: Adhesive junctions
AMD: Adenosylmethionine decarboxylase
AMPK: Adenosine 5'-monophosphate-activated protein kinase
ASA: L-aspartate-β-semialdehyde
AUBPs: AU-rich element binding proteins
AUF1: AU-rich element-RNA binding factor 1
AZ1: Antizyme 1
AZIN1: Antizyme inhibitor 1
CASDC: Carboxyspermidine decarboxylase
CASDH: Carboxyspermidine dehydrogenase
CoA: Acetyl-coenzyme A
Cx43: Connexin 43
C/EBPα: CCAAT enhancer binding protein α
dcSAM: Decarboxylated S-adenosylmethionine
DHH: Deoxyhypusine hydroxylase
DHS: Deoxyhypusine synthase
DSS: Dextran sulfate sodium
FGF21: Fibroblast growth factor 21
GABA: γ-Aminobutyric acid
GSH: Glutathione
GST: Glutathione-S-transferase
HuR: Human antigen R
IECs: Intestinal epithelial cells
LPS: Lipopolysaccharide
MdtJI: Spermidine transporter protein
MTA: Methylthioadenosine
mTOR: Mammalian target of rapamycin
NCPAH: N-carbamoylputrescine aminohydrolase
NPM: Nucleophosmin
ODC1: Ornithine decarboxylase 1
PAO: Polyamine oxidase
PLP: Pyridoxal phosphate
PotABC: ATP-binding cassette protein
PTPN2: Protein tyrosine phosphatase non-receptor type 2
RBPs: RNA-binding proteins
ROS: Reactive oxygen species
SAM: S-adenosylmethionine
SAT: Spermidine acetyltransferase
SMO: Spermine oxidase
SMS: Spermine synthase
SOD: Superoxide dismutase
SRM: Spermidine synthase
SSAT: Spermidine/spermine-N^1^-acetyltransferase
STIM1: Stromal interaction molecule 1
TJs: Tight junctions
TRPC1: Transient receptor potential channel 1
YdcW: γ-Aminobutyraldehyde dehydrogenase
YgjG: Putrescine transaminase
ZO: Zonula Occludens
3'UTR: 3'Untranslated region
